# Neuro-inflammation, blood-brain barrier, seizures and autism

**DOI:** 10.1186/1742-2094-8-168

**Published:** 2011-11-30

**Authors:** Theoharis C Theoharides, Bodi Zhang

**Affiliations:** 1 Molecular Immunopharmacology and Drug Discovery Laboratory, Department of Molecular Physiology and Pharmacology, Tufts University School of Medicine, 136 Harrison Avenue, Boston, MA, USA; 2 Departments of Biochemistry, Tufts University School of Medicine, 136 Harrison Avenue, Boston, MA, USA; 3 Departments of Internal Medicine, Tufts University School of Medicine and Tufts Medical Center, 136 Harrison Avenue, Boston, MA, USA; 4 Departments of Psychiatry, Tufts University School of Medicine and Tufts Medical Center, 136 Harrison Avenue, Boston, MA, USA

**Keywords:** Autism, Blood-Brain Barrier, Mast cells, Neuroinflammation, Flavonoids

## Abstract

Many children with Autism Spectrum Diseases (ASD) present with seizure activity, but the pathogenesis is not understood. Recent evidence indicates that neuro-inflammation could contribute to seizures. We hypothesize that brain mast cell activation due to allergic, environmental and/or stress triggers could lead to focal disruption of the blood-brain barrier and neuro-inflammation, thus contributing to the development of seizures. Treating neuro-inflammation may be useful when anti-seizure medications are ineffective.

## Background

Autism Spectrum Disorders (ASD) are pervasive neurodevelopmental disorders affecting almost 1/100 children and are characterized by difficulties in social skills, concentration, language, learning and stereotypic behaviors [1-3]. About 22-30% of children with ASD also develop seizures with no specific underlying pathology, and no obvious or classic EEG changes [1,4-7]. These rates of seizures in ASD are about ten times higher than that in the general population [8]. This high rate is not found in other neurologic diseases such as schizophrenia [9]. Alterations in architecture of cortical neurons were recently reported in autism [10] and may contribute to seizures.

Epileptic symptoms in children with ASD were recently considered to be related to immune-mediated pathogenesis [11]. In fact, ASD are associated with some immune dysfunction, such as elevated antibody levels directed against the fetal brain [12-15] suggesting BBB disruption. A recent paper from the Autism Phenome Project reported that 42% of 3 year old children with ASD and controls had plasma antibodies against GABAergic cerebellar neuron proteins, but those control children had high scores on the Child Behavior Check list, suggesting that they may constitute a susceptible subtype for ASD [16]. Moreover, IL-6 expression was elevated in the brains of ASD patients [17], while increased serum IL-6 was linked to the expression of an autistic phenotype in mice [18,19].

There is new evidence that the environment contributes significant to ASD pathogenesis [20]. Many ASD patients suffer from food allergies [21]. Moreover, 25% of ASD children have "allergic-like" symptomatology [22], but often without positive skin or RAST tests, suggesting mast cell activation by non-allergic triggers [23], including mercury [24]. Many studies delineate the importance of mast cells in both innate and acquired immunity [25], as well as in inflammation [26]. Substances originating in the gut or the brain can trigger mast cells to release mediators that could disrupt the gut-blood barrier and blood-brain barrier (BBB), thus contributing to the pathogenesis of autism [27]. Many mediators, such as IL-6 [28], can be released from mast cells "selectively" [29], making histological evaluation impossible. More importantly, mast cells have been implicated in the pathogenesis of seizures. One study using a mouse model showed that the non-allergic mast cell trigger compound 48/80 significantly increased the rate of seizures in mice induced by electric shock, and this effect was eliminated in mast cell-depleted mice [30]. Moreover, the mast cell trigger neurotensin (NT) [31] can facilitate *N*-Methyl-D-aspartate (NMDA)-induced excitation of cortical neurons [32] and seizure activity in rodents [33]. NT was increased in young children with autism [34], and was proposed as a possible therapeutic target for autism also due to its ability to induce neurotoxicity [35].

Children with mastocytosis, a spectrum of diseases that present with skin allergies and diarrhea, also complain of learning disabilities, hyperactivity and difficulty focusing ("brain fog"), reminiscent of ASD [36,37]. In fact, children with mastocytosis have a 10-fold higher prevalence of ASD (1/10 children) than that reported for the general population (1/100 children) [38]. Interestingly, mastocytosis patients also have high serum IL-6 levels [39,40] and develop seizures [41]. Also, a solitary mastocytoma produced symptoms mimicing seizures [42].

## Hypothesis

**Figure 1:**
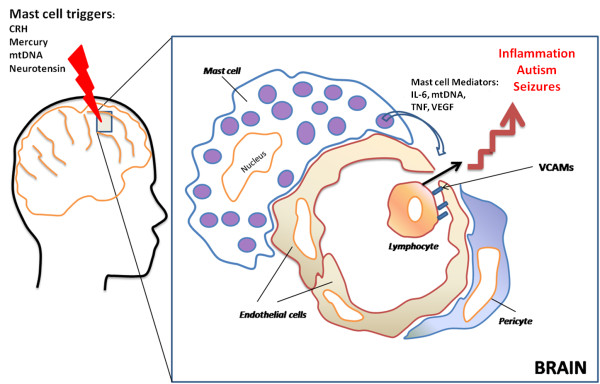
**Mast cells are located perivascularly close to nerve endings and regulate blood-brain barrier permeability**. Upon stimulation by allergic and non-immune triggers (e.g, CRH, neurotensin, mercury, mitochondrial (mt) DNA), mast cells release vasodilatory and inflammatory molecules (IL-6, mtDNA, TNF and VEGF), some of which increase the expression of vascular endothelial cell adhesion molecules (VCAMs) and permit exit of circulating lymphocytes in the brain. Focal brain inflammation could then contribute to or exacerbate seizures.

Immune dysfunction and inflammation appear to alter BBB integrity [43,44]. Recent evidence indicates that the integrity of the BBB, especially leukocyte endothelial adhesion may also be involved in the pathogenesis of epilepsy [45], a phenomenon described as "immunology: barrier to electrical storms" [46]. Mast cells were considered as the "immune gate to the brain" [47]. ASD patients are prone to stress [48], and prenatal stress has been linked to risk of autism [49]. The brain, especially the hypothalamus, contains many mast cells critically located around the BBB, and that stress activates brain mast cells leading to BBB disruption [50]. Moreover, corticotropin-releasing hormone (CRH), secreted under stress, can activate mast cells [51] and is responsible for mast cell-dependent BBB disruption [50,51]. The possible involvement of mast cells is further supported by the ability of histamine-1 receptors to augment seizures [52]. Brain mast cells also contribute to the pathogenesis of migraine headaches [53] that increase the likelihood of seizures [54]. Local activation of brain mast cells could lead to focal disruption of the BBB, permitting focal neuro-inflammation that could become an epileptogenic site (Figure 1). This process could worsen by activation of Fcgamma receptors (FcγRI) on neurons that could contribute to brain cell death after injection of the epileptogenic kainic acid [55]. Moreover, Fcepsilon receptors (FcεRI), typically thought to be expressed only by mast cells and basophils, were recently identified on neurons [56] implying that allergic triggers may even affect the neurons directly, once the BBB has been disrupted to permit entry of immunoglobulins. A recent publication reported that increased serum level of "high mobility group box 1 protein (HMGB1) in young autistic patients [57]. HMGB1 is released from neurons following neurotoxicity [58] and it was recently shown to constitute a pro-seizure pathway through activation of toll-like receptors (TLR-4) in mice [59]. We recently showed that mast cell activation leads to mitochondrial translocation to the cell surface [60], and secretion of extracellular mtDNA [34]. We also showed that serum of children with autism had increased levels of extracellular mitochondrial DNA [61]. Damaged Associated Molecular Patterns (DAMPs), which include mitochondrial DNA, were reported to be released from damaged cells in trauma patients and activate TLR leading to auto-inflammation [62]. Mitochondrial DNA was also reported to be directly neurotoxic and alter behavior in mice [63].

Anticonvulsant medications often are ineffective in both ASD and mastocytosis patients with seizures [64]. It would, therefore, be important to use treatment approaches directed to the core symptoms of ASD and/or brain mast cell activation and inflammation [23]. Use of select, natural, flavonoids, may be useful because of their anti-oxidant and anti-inflammatory ability [65]. Luteolin is a flavone contained in chamomile and chrysanthemum. Increasing evidence indicates that luteolin has potent antioxidant, free radical scavenger, anti-inflammatory and mast cell inhibitory activity [66]. In addition, luteolin inhibits microglia IL-6 release [67,68], as well as mimics the activity of brain-derived neurotrophic factor (BDNF) [69]. Luteolin also inhibits autistic-like behavior in mice [70]. Luteolin also inhibits mast cell-dependent stimulation of activated T cells [71], as well as activated peripheral blood mononuclear cells from patients with the brain inflammatory disease multiple sclerosis [72]. Moreover, the structural flavone analogue, quercetin, found in citrus pulp and peels, is also a potent mast cell inhibitor [73] and has anti-seizure activity [74], as does its natural glycoside rutin [75]. Luteolin may, therefore, be useful for the treatment of neuroinflammation, including seizures in ASD children, especially if administered in formulations that permit sufficient oral absorption.

## Implications

In conclusion, evidence reviewed above indicates a possible association between neuroinflammation, mast cell activation and seizures, through secretion of pro-inflammatory mediators and regulation of the BBB permeability. Mast cell function inhibitors, especially those blocking the effect of NT, such as luteolin, may serve as novel therapeutic agents for the treatment of autism and related seizures.

## Competing interests

TCT is the inventor of US patents No. 6,624,148; 6,689,748; 6,984,667, and EPO 1365777, which cover methods and compositions of mast cell blockers, including flavonoids, US patents 7,906,153 and 12/861,152 (allowed on September, 22, 2011) for treatment of neuroinflammatory conditions, as well as US patent applications No.12/534,571 and No.13/009,282 for the diagnosis and treatment of ASD. TCT is also the inventor of the dietary supplement, NeuroProtek^®^, which has the US trademark No 3,225,924.

## Authors' contributions

TCT and BZ prepared, read, and approved this manuscript.
